# Epicardial Adipose Tissue Volume Assessment in the General Population and CAD-RADS 2.0 Score Correlation Using Dual Source Cardiac CT

**DOI:** 10.3390/diagnostics15060681

**Published:** 2025-03-10

**Authors:** Federica Dell’Aversana, Renato Tuccillo, Alessandro Monfregola, Leda De Angelis, Giovanni Ferrandino, Carlo Tedeschi, Fulvio Cacciapuoti, Fabio Tamburro, Carlo Liguori

**Affiliations:** 1 Department of Precision Medicine, University of Campania “L. Vanvitelli”, 80138 Naples, Italy; fe.dellaversana@gmail.com (F.D.); renatotuccillo96@gmail.com (R.T.); 2 Division of Radiology, University of Napoli “Federico II”, 80131 Naples, Italy; dr.alessandromonfregola@gmail.com (A.M.); ledadeangelis1@gmail.com (L.D.A.); 3 Department of Radiology, Ospedale del Mare-ASL Napoli 1, 80147 Naples, Italy; gianni.ferrandino90@gmail.com (G.F.); fabio.tamburro@aslnapoli1centro.it (F.T.); 4 Operational Unit of Cardiology, Presidio Sanitario Intermedio Napoli Est, ASL-Napoli 1 Centro, 80144 Naples, Italy; carlo.tedeschi@hotmail.it; 5 Department of Cardiology, Azienda Ospedaliera di Rilievo Nazionale Antonio Cardarelli, 80131 Naples, Italy; fulvio.cacciapuoti@aocardarelli.it; 6 Department of Radiology, San Giovanni Bosco-ASL Napoli 1, 80144 Naples, Italy

**Keywords:** epicardial adipose tissue (EAT), coronary computed tomography angiography (CCTA), coronary artery disease reporting and data system (CAD-RADS), coronary artery disease (CAD), coronary artery calcification (CAC)

## Abstract

**Objectives:** Our study aims to investigate the correlation between epicardial adipose tissue (EAT) volume assessed with non-contrast cardiac CT (NCCCT) and sex, age, coronary artery disease reporting and data system (CAD-RADS 2.0) categories, and coronary artery calcification (CAC) extent. The secondary aim is to establish the average values of EAT in a population considered healthy for coronary artery disease (CAD). **Materials and Methods:** We retrospectively analyzed patients who underwent coronary computed tomography angiography (CCTA) at our institution from January 2023 to August 2024. The CAD-RADS 2.0 scoring system was applied to assess the extent of CAD; CAC extent was quantified according to the Agatston score. EAT was segmented semi-automatically in NCCCT images, and its volume was subsequently measured. Correlation analyses between EAT volume, sex, patient age, CAC, and CAD-RADS categories were conducted. **Results:** A total of 489 consecutive patients met the inclusion criteria (63.96 ± 12.18 years; 214 females). The mean EAT volume ± SD in those categorized as CAD-RADS 0 (57.25 ± 15.45 years, 120 patients) was 117.43 ± 50.30 cm^3^: values were higher in men (121.07 ± 53.31 cm^3^) than in women (114.54 ± 47.98 cm^3^). EAT volumes positively correlated with age, male sex, CAD severity, and CAC scores. **Conclusions:** According to our results, males in all CAD-RADS categories have a greater amount of EAT than females. A positive correlation between the volume of EAT and factors such as age (*p* = 0.003), CAD-RADS categories (*p*: 0.004), and coronary calcium score (*p* = 0.0001) with a strong influence exerted by sex was demonstrated. Our results reinforce the observation that higher EAT volumes are associated with a more severe coronary artery disease.

## 1. Introduction

Epicardial adipose tissue (EAT) is a fat deposit with unique characteristics that distinguish it from any other type of adipose tissue, both in its composition and anatomical location. EAT is a white adipose tissue mostly located under the visceral layer of the pericardium, in the atrioventricular and interventricular sulci, and lies directly above the myocardium [1].

Interestingly, EAT shares its blood supply with the myocardium, receiving perfusion from branches of the epicardial coronary arteries, which are embedded within the EAT along most of their course. Furthermore, EAT provides structural support preventing coronary arteries from twisting, stores triglycerides which produce energy promptly used by myocardial cells, and is a major source of mesenchymal stem cells to regenerate cardiomyocytes [2,3,4].

Numerous studies have investigated EAT’s role in the pathogenesis of different cardiac pathologies such as atrial fibrillation and heart failure. Regional accumulation of EAT in the atria and ventricles can exert structural and electrical remodeling, and a strong association between a high burden of epicardial fat with heart failure with preserved ejection fraction (HFpEF) has been demonstrated [3,5,6].

Moreover, a direct correlation between increased EAT volume and both the presence and the severity of coronary artery disease (CAD) has also been demonstrated [7,8].

Coronary artery disease reporting and data system (CAD-RADS) is a standardized classification system of CAD for patients undergoing coronary computed tomography angiography (CCTA). In 2022, the updated version was released, CAD-RADS 2.0, which includes some novelties such as a grading scale for plaque burden and ischemia detection in addition to CAD severity grading system based on the most severe stenosis [9].

Since CCTA has emerged as a powerful modality to exclude CAD in symptomatic patients with a low-to-intermediate risk of CAD, it has entered several guidelines as a first-line test (recommendation of class I). Numerous trials and registries have been conducted to evaluate the prognostic significance of the CAD-RADS classification, and its utility has been consistently validated. In fact, with the SCOT-HEART [10] and PROMISE trials [11], the usefulness of CCTA has been confirmed in addition to the standard of care and as an alternative to the functional test. Moreover, it has been demonstrated that the risk of fatal and non-fatal MI is increased in patients with higher CAD-RADS scores [12].

Several studies have demonstrated that CCTA represents the gold standard non-invasive modality to measure EAT because of its high spatial resolution. Multislice computed tomography (MSCT) allows to simultaneously measure the EAT volume and the CAD-RADS category, and both parameters can provide an excellent prediction of the risk of cardiovascular events [13,14].

However, to the best of our knowledge, a correlation between these two independent parameters has not been performed yet. To fill this gap in the literature, our study aims to establish the average values of EAT volume assessed with non-contrast cardiac CT (NCCCT) and to investigate the correlation between EAT NCCCT-assessed volumes and sex, age, CAD-RADS 2.0 categories, and coronary artery calcification (CAC) extent.

The secondary target was to establish the average values of EAT in a population considered healthy for CAD.

## 2. Materials and Methods

### 2.1. Patients Selection

This retrospective study included patients who underwent CCTA between January 2023 and August 2024 at our radiology department in which the CAD-RADS 2.0 scoring system was applied to assess the extent of CAD. CCTA images were retrospectively obtained from the picture archiving and communication system (PACS). The study received approval from the institutional review boards. Before CCTA, all patients herein considered were informed about the possible use of their data for study purposes and gave their consent. Data were anonymized prior to analysis to ensure confidentiality.

Patients were enrolled according to the following inclusion criteria: (i) availability of the NCCCT dataset for quantitative measurements, (ii) availability of demographic and biometric parameters, and (iii) availability of cardiovascular medical history.

**Figure 1:**
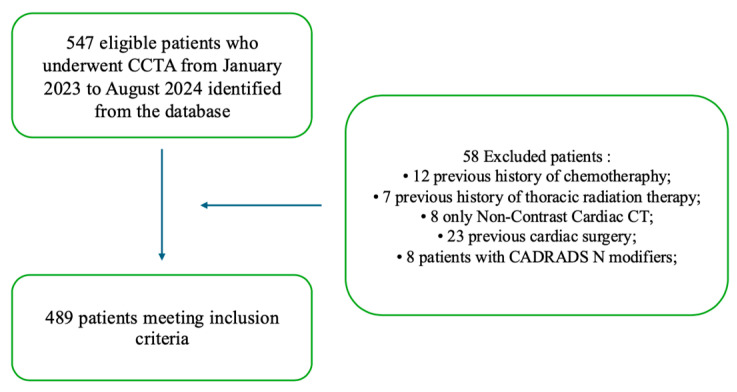
Enrollment flowchart of the study.

Exclusion criteria were as follows: (i) previous surgical coronary revascularization, (ii) previous cardiac surgery, (iii) previous chemotherapy, (iv) previous thoracic radiation therapy, and (iv) CAD-RADS N category. The enrollment flowchart is shown in Figure 1.

The main aim was to investigate the correlation between EAT volume assessed with NCCCT and sex, age, CAD-RADS 2.0 categories, and CAC extent. The secondary aim was to establish the average values of EAT volume in a population considered healthy for CAD and divide the study cohort into two populations based on the presence or absence of CAD to investigate the presence of statistically significant differences between the two subgroups.

### 2.2. Coronary Computed Tomography Angiography: Image Acquisition

CCTA exams were conducted in hemodynamically stable patients using a third-generation dual-source dual-energy computed tomography scanner (Somatom Drive, Siemens Healthineers, Forchheim, Germany). Those with a history of allergic reaction to iodinated contrast media, impaired renal function, and inability to hold their breath for 10 s were not tested.

All rhythms were accepted for scanning and a calcium score evaluation, used for EAT quantification, with a low-dose, high-pitch ECG-triggered non-contrast cardiac scan at 65% of the R-R interval at end-inspiratory breath hold was performed. The acquisition encompassed the entirety of the heart, extending from the level of the carina down to the apex of the heart, ensuring comprehensive coverage of the cardiac region. The cardiac scan was performed in spiral mode, with a pitch value of 3.2, the tube current was 64 reference milliampere-seconds per rotation, and the tube voltage was set to 100 kilovolts peak. The matrix size used for image reconstruction was 512 × 512 with a field of view (FOV) of 250 mm centered on the heart and a slice thickness of 3.0 mm. ADMIRE iterative reconstructions and a medium sharp kernel (Sa36) for soft tissue were used in every exam. The heart rate during scan acquisition was reported on the reconstructed CT series images. A total of 5 mg sublingual nitroglycerin was administered, unless contraindicated, in the CT suite before the scan by the supervising physician to provide transient coronary dilatation.

For subsequent coronary evaluations, intravenous beta-blocker (metoprolol) administration was evaluated on a case-by-case basis based on the indications of the referring clinician. Patients with a heart rate > 65 beats per minute (bpm) were administered 5 mg of intravenous metoprolol.

After NCCCT acquisition, a high-concentration iodinated contrast medium (370–400 mgI/mL) was injected to obtain an iodine delivery rate (IDR) of 2 gI/s through the antecubital vein at a rate of 5 mL/s through a double-piston power injection followed by the injection of 40 mL of saline solution at the same rate. Then, a retrospectively modulated ECG-gated cardiac arterial phase CCTA was acquired. The region of interest (ROI) was positioned in the ascending aorta and an opacification greater than 150 HU automatically triggered scan acquisition. The scanner parameters were based on the patient’s body mass index (BMI): the rotation time was 270 milliseconds, the collimation was 128 × 0.625 mm, the tube voltage was 100–120 kVp, and the tube current was 200–300 mAs. The axial image reconstruction was performed at a 0.4 mm slice thickness with iterative methods. All scans were supervised by a physician with subspecialty training in cardiovascular imaging.

### 2.3. Coronary Computed Tomography Angiography: Images Analysis

All CCTA images were imported to the postprocessing workstation (Syngovia, Siemens Healthineers). Two radiologists with 20 and 5 years of experience in cardiac imaging analysis (C.L., G.F.), respectively, visually inspected both the cross-sections and the longitudinal reconstructed images to detect coronary plaques or anatomical anomalies. Among the CAC scoring methods, the Agatston score was applied in each patient to quantify the amount of calcified plaque burden [15]. The presence of atherosclerotic plaques and stenosis grading were evaluated on coronary segments with a diameter greater than 1.5 mm in accordance with the 18-segment model recommended by the Society of Cardiovascular Computed Tomography (SCCT) [16].

CAD-RADS stenosis categories were assessed on the basis of the degree of maximal coronary stenosis as follows: CAD-RADS 0 was defined as presenting no plaques or CAD, CAD-RADS 1 as presenting a maximal coronary stenosis of 1–24%, CAD-RADS 2 as presenting a maximal coronary stenosis of 25–49%, CAD-RADS 3 as presenting a maximal coronary stenosis of 50–69%, CAD-RADS 4A as presenting a maximal coronary stenosis of 70–99%, CAD-RADS 4B as presenting a maximal coronary stenosis of the left main >50% or a three-vessel disease, CAD-RADS 5 as presenting a maximal coronary stenosis of 100% or a total occlusion of the vessel lumen according to the CAD-RADS 2.0 consensus document. The overall amount of coronary plaque or “P” category was assigned in relation to CAC as follows: P1 was defined as presenting a mild amount of coronary plaque CAC 1-100, P2 as presenting a moderate amount of coronary plaque CAC 101-300, P3 as presenting a severe amount of coronary plaque CAC 301-999, and P4 as presenting extensive amount of coronary plaque CAC > 1000 [9]. 

### 2.4. Coronary Computed Tomography Angiography: Epicardial Adipose Tissue Measurement

**Figure 2:**
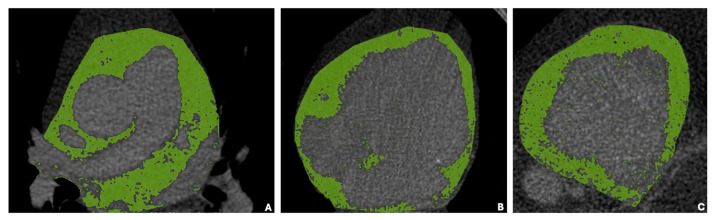
(**A**–**C**) Non-contrast cardiac CT axial views at three different anatomical levels of the same patients (EAT volume: 414 cm^3^). (**A**) shows pericardial contours drawn at the most superior slice, at the bifurcation of the main pulmonary artery; (**B**) pericardial contours at the medial slice and (**C**) at the most inferior slice, where the posterior descending coronary artery in the inferior atrioventricular groove is last seen. The segmentation process of the epicardial adipose tissue (green areas) was completed using the quantification software.

The total EAT was considered as the visceral adipose tissue found between the myocardium and the visceral layer of the pericardium (Figure 2). As previously described in the literature [17], upper anatomic boundaries for EAT volume measurement included the pulmonary artery bifurcation, the mid left atrium, and the aortic root. Lower anatomic boundaries were the left ventricular apex and the diaphragm. The whole EAT volume was independently manually traced by two operators (L.D., A.M.) on NCCCT with a quantitative semi-automated procedure using 3 mm thick axial slices used for calcium scoring. Depending on heart size, the number of slices traced manually ranged from 6 to 9 in each patient.

The computer software (Multimedia Reading, Syngo.via (VBOS_HF02), Siemens Healthineers, Erlangen, Germany) then automatically interpolated and traced the visceral pericardium in all slices interposed between the manually traced slices. The sagittal and coronal views were used to assess the accuracy of each automatic segmentation. Threshold attenuation values of −30 HU to −190 HU were adopted to identify fat voxels. Then, the selected volume was extracted in cubic centimeters (cm^3^). The interobserver and intraobserver variability was <5%.

### 2.5. Statistical Analysis

Interobserver reproducibility among the radiologists involved in EAT volume NCCT image contouring was assessed with the coefficient of variation calculation. The intraclass correlation coefficient (ICC) for continuous variables was used to define interobserver variability. Lin’s concordance correlation coefficient (CCC) was considered an index of CT; segmentation reproducibility between EAT volume measurements and perfect agreement was reached with a CCC value greater than 0.990.

The Shapiro–Wilk test was used to assess the normality of the data distribution. Variables with a normal distribution were expressed as the mean ± standard deviation (SD), while non-normally distributed variables were reported as median and interquartile range (IQR). Categorical variables are reported as frequencies and percentages. Appropriate statistical tests were applied for comparisons based on the parametric or nonparametric nature of the variables. Categorical variables were compared using the chi-square test. Univariate linear regression analysis was used to evaluate the relationship between log-transformed epicardial adipose tissue (EAT) volume and various clinical variables in patients with and without coronary artery disease (CAD). The clinical variables included sex, age, BMI, coronary artery calcium (CAC), and traditional cardiovascular risk factors such as smoking, hypertension, diabetes, and dyslipidemia. Since EAT volume was not normally distributed, a logarithmic transformation (logVol) was applied to normalize the residuals and ensure robust statistical modeling. The *p*-values were used to determine statistical significance, with a threshold of *p* < 0.05 indicating significant associations.

The coefficients indicated the direction and strength of the relationship between the independent variables (sex, age) and the dependent variable (log-transformed EAT volume).

A multivariate logistic regression model was constructed to assess the relationship between coronary artery disease (CAD) and various clinically relevant variables. Variables included in the model were selected based on their significance in the univariate Pearson correlation analysis and their variance inflation factor (VIF < 2) to mitigate multicollinearity. The model included log-transformed EAT volume, age, and coronary artery calcium (CAC) scores. Odds ratios (OR) and 95% confidence intervals (CI) were calculated to interpret the likelihood of CAD associated with each predictor.

Statistical analyses were performed using SPSS statistical analysis software (IBM SPSS statistics for macOS, Version 29.0.2.0 Inc., Chicago, IL, USA).

## 3. Results

As shown in Table 1, a total of 489 consecutive patients met the inclusion criteria of this study. Among these, 275 (56.2%) were male and 214 (43.8%) were female, with ages ranging from 19 to 90 years. The Shapiro–Wilk test demonstrated the non-normal distribution of age, BMI, EAT volume, and CAC (*p*-value < 0.001); for this reason, these variables are summarized using the median and interquartile range (IQR). The median age of the general population was 64 years (interquartile range [IQR]: 16). When broken down by sex, women were older on average, with a median age of 67 years (IQR: 15) compared to men whose median age was 63 (IQR: 16). The median body mass index (BMI) was 26 (IQR: 4). Regarding epicardial adipose tissue (EAT), the median volume was 133 cm^3^ (IQR: 73). The median calcium Agatston score was 60 (IQR: 324), indicating a wide variability in coronary calcium deposition within the cohort. In terms of cardiovascular risk factors, 179 patients (36.6%) were smokers, 144 (29.4%) had diabetes mellitus, 257 (52.6%) had high blood pressure, and 218 (44.6%) had dyslipidemia. Additionally, 108 patients (22.1%) reported a family history of heart disease.

In Table 2, the study cohort is categorized according to the CAD-RADS 2.0 stenosis grading system, along with modifiers for coronary artery metallic stents (S) and high-risk plaques (HRP). The median EAT volume expressed in cubic centimeters and IQR for each CAD-RADS category is provided for the entire population, as well as for men and women separately.

This table offers a detailed overview of EAT volume across the various CAD-RADS categories, shedding light on both gender differences and the extent of EAT variation in relation to coronary artery disease severity. In terms of patient distribution, the largest proportion of individuals falls under CAD-RADS 0 (24.5%), indicating the absence of coronary artery disease. At the other end of the spectrum, CAD-RADS 5, which represents severe coronary artery disease, accounts for 5.7% of the population. Additionally, the table includes important diagnostic modifiers such as HRP (high-risk plaque) and S (stents) which reflect specific clinical conditions.

When examining the gender distribution, a clear trend emerges, with women more prevalent in the lower-risk group (CAD-RADS 0, 55.9%) while men dominate the higher-risk categories, particularly CAD-RADS 5 (82.1%). This pattern is consistent with gender-related differences in the progression of coronary artery disease, with men more frequently represented in the more severe stages of the condition.

Regarding EAT volume trends, there is a noticeable increase in EAT volume as CAD-RADS categories increase in severity, peaking in CAD-RADS 5 with a median volume of 149 cm^3^ and an interquartile range (IQR) of 72 cm^3^. Women tend to have lower EAT volumes in the early stages of coronary disease, though their variability decreases in advanced stages. In contrast, men consistently exhibit higher EAT volumes, especially in the more severe CAD-RADS 4 and 5 categories. This suggests a potential link between increased visceral fat and the progression of advanced coronary artery disease in men.

Focusing on modifiers, the HRP group shows a median EAT volume of 142 cm^3^ (IQR 71 cm^3^), indicating a significant accumulation of epicardial fat associated with high-risk plaque. This further supports the idea that EAT may play a crucial role in identifying patients at higher risk of adverse cardiovascular events.

To evaluate the relationship between log-transformed EAT volume and CAD-RADS categories, a linear regression analysis was performed. Log-transformed EAT volume (logVol) was the dependent variable and the CAD-RADS category was the independent variable, treated as a continuous variable reflecting increasing severity of coronary artery disease. The linear regression analysis revealed a significant association between CAD-RADS categories and log-transformed EAT volume (*p*-value: 0.004). This *p*-value indicates that the relationship between CAD-RADS severity and EAT volume is statistically significant (*p* < 0.05). Thus, as the CAD-RADS category increases (indicating more severe coronary artery disease), the epicardial fat volume also tends to increase.

**Figure 3:**
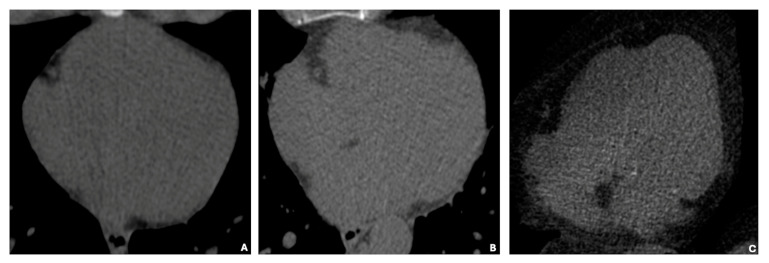
(**A**–**C**) show basal cardiac axial scans obtained through non-contrast computed tomography (NCCT) of individuals with varying epicardial adipose tissue (EAT) volumes. The images represent three distinct categories: low EAT volume (35 cm^3^) (**A**), average EAT volume (125 cm^3^) (**B**), and high EAT volume (414 cm^3^) (**C**).

In Figure 3, basal cardiac axial scans obtained through non-contrast computed tomography (NCCT) illustrate three distinct categories of EAT volume: low (35 cm^3^), average (125 cm^3^), and high (414 cm^3^). The images visually depict the increasing EAT volume across these categories.

**Figure 4:**
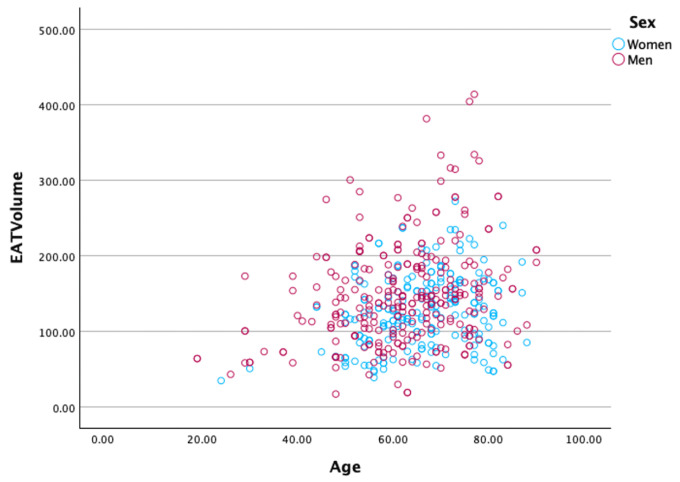
Scatter plot depicting the distribution of epicardial adipose tissue (EAT) volume frequency (y-axis) in women and men, stratified by age (x-axis). This visualization highlights the relationship between age and EAT volume for each sex, providing a comparative perspective on how EAT accumulation differs between men and women across various age groups.

Additionally, Figure 4 presents scatter plots illustrating the distribution of EAT volume in relation to age and sex. The positive correlation between EAT volume and age is evident, with older individuals exhibiting higher EAT volumes. Furthermore, men generally have higher EAT volumes than women across the entire age range, further emphasizing the sex-based differences in EAT accumulation.

CAC was measured in 399 patients (81.6% of the cohort), and classified in four plaque burden categories from P0 to P4. Table 3 presents the median epicardial adipose tissue (EAT) volume and its interquartile range (IQR) across different coronary artery calcium (CAC) categories in the general population, as well as stratified by sex (women and men).

In the general population, the EAT volume shows a gradual increase with higher CAC categories, from 126 cm^3^ in patients with no calcification (CAC 0) to 145.0 cm^3^ in those with severe calcification (P4 > 1000).

When examining gender-specific trends, men consistently exhibit higher EAT volumes across all CAC categories. Their EAT volume increases steadily with rising CAC, peaking at 154 cm^3^ in P4 (>1000). Conversely, women show a different trajectory: while EAT volume increases up to P2 (101–300), peaking at 147.0 cm^3^, it unexpectedly declines in the most severe CAC category (P4 > 1000, median 114.83 cm^3^). This divergence suggests that the relationship between EAT volume and coronary calcification may be influenced by gender-specific factors, such as hormonal differences, body fat distribution, or metabolic variations.

Additionally, the variability in EAT volume (as indicated by the interquartile range, IQR) is generally greater in men, particularly in intermediate CAC categories (P1 and P3). This variability could reflect a broader spectrum of cardiovascular risk profiles among male patients.

A univariate linear regression analysis (Table 4) evaluated the relationship between log-transformed epicardial adipose tissue (EAT) volume and various clinical variables in patients with and without coronary artery disease (CAD). The clinical variables included sex, age, BMI, coronary artery calcium (CAC), and traditional cardiovascular risk factors such as smoking, hypertension, diabetes, and dyslipidemia.

The general population was divided into two groups, 120 patients with CADRADS 0 were considered healthy for CAD and the remaining 369 were classified as positive for coronary atherosclerotic disease. Age, BMI, CAC, smoking, hypertension, and dyslipidemia showed strong, significant associations with EAT volume in individuals without CAD. Notably, smoking had a negative association with EAT volume in the non-CAD group, indicating a complex interaction between smoking habits and visceral fat distribution. In patients with CAD, diabetes mellitus emerged as the only significant predictor of EAT volume (*p* = 0.043), emphasizing the role of metabolic dysfunction in epicardial fat accumulation in the context of established coronary artery disease. Traditional risk factors like age, BMI, and CAC lost their predictive power in the CAD group, suggesting that epicardial fat dynamics may shift once coronary disease is present. Variables such as sex and hypertension were not significant in the CAD group, though they showed trends toward significance, particularly for sex (*p* = 0.061).

**Figure 5:**
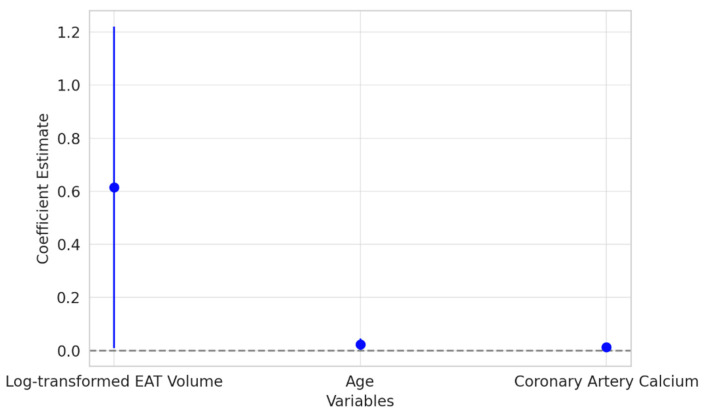
Graphic in the figure illustrates the coefficients from the multivariate logistic regression model along with their 95% confidence intervals for the variables: log-transformed EAT volume, age, and coronary artery calcium (CAC). The log-transformed EAT volume shows a strong positive association with coronary artery disease (CAD). The confidence interval does not cross zero, indicating that higher levels of epicardial fat significantly increase the likelihood of CAD.

The results of the multivariate logistic regression analysis (Figure 5) revealed that log-transformed EAT volume was a significant independent predictor of CAD. Patients with higher EAT volumes were more likely to have CAD, underscoring the potential role of epicardial fat as an active contributor to the development of coronary artery disease. This association persisted even after adjusting for other traditional risk factors, emphasizing the clinical relevance of EAT in cardiovascular risk assessment.

**Figure 6:**
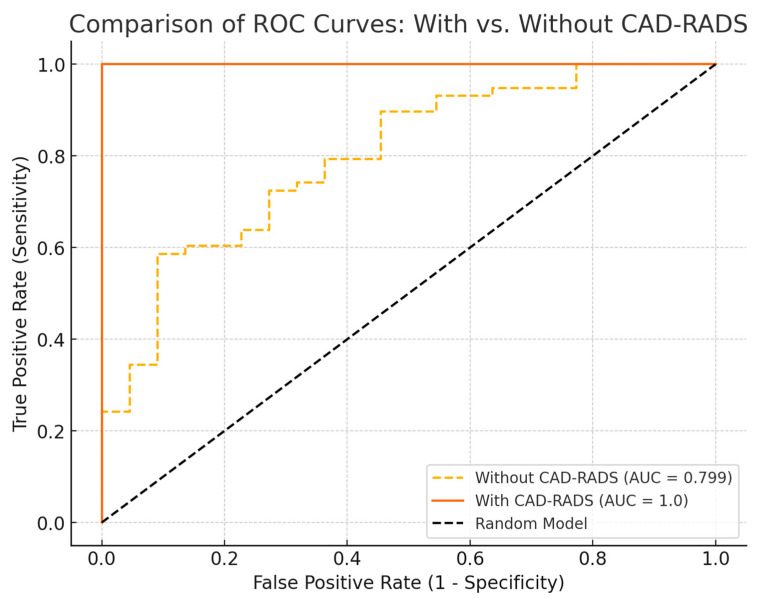
Comparison of ROC curves for logistic regression models with and without CAD-RADS. The model including CAD-RADS (AUC = 1.0) shows perfect discrimination, while the model excluding CAD-RADS (AUC = 0.799) retains good predictive power but with reduced accuracy.

To further investigate the predictive value of EAT and CAC independently, we conducted an additional logistic regression analysis excluding CAD-RADS as a predictor (Figure 6). This model demonstrated an AUC-ROC score of 0.799, indicating a good but not perfect discriminatory ability between patients with and without CAD. The classification performance revealed a high recall of 94.8% for CAD-positive patients but a lower recall of 22.7% for CAD-negative patients, suggesting that EAT volume and CAC contribute significantly to CAD prediction but do not fully capture the discriminative power provided by CAD-RADS.

The original logistic ridge regression model, which included CAD-RADS, demonstrated a perfect discriminatory ability with an AUC-ROC score of 1.0, indicating that the combination of EAT volume, coronary artery calcium (CAC) score, and CAD-RADS category effectively distinguishes between patients with and without CAD. Classification metrics showed 100% precision, recall, and F1-scores for both groups, supported by a confusion matrix with 111 true negatives (non-CAD patients correctly classified) and 288 true positives (CAD patients correctly classified), with no misclassification.

Statistical comparisons between CAD and non-CAD groups revealed significant differences: EAT volume showed a t-statistic of 3.99 with a *p*-value < 0.001, indicating significantly higher EAT volumes in CAD patients. CAC scores had a t-statistic of 6.37 with a *p*-value < 0.001, demonstrating a strong association between coronary calcium and CAD. CAD-RADS categories exhibited the strongest difference, with a t-statistic of 20.19 and a *p*-value < 0.001, reflecting its robust predictive power.

Coronary artery calcium (CAC) also displayed a positive relationship with CAD, though the effect was less pronounced compared to the EAT volume. As expected, CAC was also a significant predictor, reflecting its established role in measuring atherosclerotic plaque burden. Interestingly, age, a well-known risk factor for cardiovascular disease, did not reach statistical significance in this model. This finding suggests that the influence of age may be overshadowed by the stronger predictive power of the EAT volume, family history, and CAC in this specific cohort. These findings highlight the potential of EAT and CAC as critical biomarkers in CAD risk stratification and reinforce the additional predictive value provided by CAD-RADS.

## 4. Discussion

In our study of adult patients with clinical indications for CCTA, we identified significant relationships between EAT volume and key cardiovascular parameters, including age, sex, CAD-RADS categories, and CAC (Agatston score). Larger EAT volumes positively correlated with age, male sex, and CAD severity, particularly with CAC scores up to 1000. Interestingly, in females, EAT volume was lower than in males across all CAD-RADS categories, underscoring potential sex-specific differences in EAT deposition and its association with CAD.

The study cohort was further divided in two groups based on the presence or absence of CAD, and subsequent univariate analyses revealed that EAT volume is significantly influenced by sex, age, BMI, CAC, and traditional cardiovascular risk factors in both populations. However, the associations were generally stronger in the CAD positive group, suggesting that epicardial fat accumulation is more closely linked to coronary artery disease in the presence of other risk factors. Multivariate analysis findings also underscored the strong association of EAT volume, CAC score, and CAD-RADS category with CAD presence, emphasizing their potential utility in clinical risk stratification and diagnostic protocols. These findings highlight the potential role of EAT volume as a marker of cardiovascular risk, particularly in patients with existing coronary artery disease.

EAT is a white adipose tissue mostly located under the visceral layer of the pericardium which also accumulates along the free wall of the right ventricle, the apex of the left ventricle, and, in some cases, can cover nearly the entire surface of the heart [1].

This direct relationship with coronary arteries is further reinforced by the absence of fascial structures separating EAT from the cardiac muscle and its endocrine potential as a source of several adipokines. EAT is a metabolically active endocrine organ composed of white adipose tissue with some functional overlapping characteristics with brown and beige ones. Its transcriptome differs from that of other visceral and subcutaneous fat depots, and it is rich in genes encoding cardioprotective adipokines with anti-inflammatory and anti-atherogenic activities whose expression is regulated by oxidative factors released by the heart during phases of myocardial oxidative stress. However, if in normal conditions EAT participates in the homeostasis of the cardiovascular system by carrying out a protective action, its function and morphology can change both with ageing and pathological conditions, becoming a site of pathological adipogenesis and of pro-inflammatory adipokines production [3,4]

Coronary artery disease reporting and data system (CAD-RADS) is a standardized classification system of CAD for patients undergoing coronary computed tomography angiography (CCTA). CAD-RADS first version was introduced in 2016 and applied in patients presenting with stable chest pain categorized based on the degree of maximal stenosis found at CCTA from 0 (no plaque or stenosis is evident) to 5 (in case of total coronary occlusion or sub-total occlusion). Each category corresponds to a clinical interpretation and suggestion for further cardiac investigation if necessary and subsequent management consideration. In 2022, the updated version was released, i.e., CAD-RADS 2.0, which includes some novelties such as a grading scale for plaque burden (P categories from P1 corresponding to a mild amount of plaque to P4 an extensive one) and ischemia detection (modifier I which indicates that an ischemia test was performed with computed tomography-fractional flow reserve (CT-FFR) or myocardial computed tomography perfusion (CTP)) [9].

Since CCTA has emerged as a powerful modality to exclude CAD in symptomatic patients with low-to-intermediate risk of CAD, it has entered several guidelines as a first-line test (class I). Numerous trials and registries have been conducted to evaluate the prognostic significance of the CAD-RADS classification, and its utility has been consistently validated. In fact, with the SCOT-HEART [10] and PROMISE trials [11], the usefulness of CCTA has been confirmed in addition to the standard of care and as an alternative to the functional test. Moreover, it has been demonstrated that the risk of fatal and non-fatal MI is increased in patients with higher CAD-RADS scores [12]. Several studies have demonstrated that CCTA represents the gold standard non-invasive modality to measure EAT because of its high spatial resolution, with both EAT volume measured with multislice computed tomography (MSCT) and CAD-RADS category providing excellent risk prediction for cardiovascular events [13,14].

In agreement with others, we found that non-enhanced ECG-gated cardiac CT scans allow for a highly reproducible EAT volumetric quantification in a non-invasive manner [18,19,20]. Although transthoracic echocardiography (TTE) is a non-invasive method with widespread use in clinical practice, it only permits the measurement of EAT thickness. However, TTE is limited by its low sensitivity in distinguishing EAT from pericardial fat, whereas CT enables the entire volume of EAT to be measured with reproducibility and accuracy [21].

EAT volume is not uniformly distributed across the cardiac surface but preferentially localizes to specific regions, including the free wall of the right ventricle, the apex of the left ventricle, the atria, epicardial coronary vessels, and cardiac appendages. These distinct anatomical locations seem to be associated with specialized functions, as evidenced by unique transcriptomic signatures tailored to the roles of the adjacent structures and a whole volumetric assessment seems to be crucial [22].

Despite the growing evidence in favor of the role played by this metabolically active tissue in the pathogenesis and outcome of the most varied cardiac pathologies [23,24,25,26] there is still no value considered normal. This is partly justified by the confusion about the nomenclature of cardiac fat depots. Often an overlap between the concepts of pericardial adipose tissue and EAT can be found [7,27,28], and some include EAT in pericardial adipose tissue (PAT) with paracardial fat, which can be found on the external surface of the parietal pericardium [29].

Our results align with the existing literature and are notable for being among the first to systematically compare EAT volumes with the updated CAD-RADS 2.0 categories. The results of the linear regression analysis demonstrate a significant positive association (*p*-value: 0.004) between EAT volume and the severity of coronary artery disease, as classified by the CAD-RADS system. Higher CAD-RADS categories correspond to a progressive increase in the mean EAT volume, peaking in CAD-RADS 5 patients (mean: 149 cm^3^; 151 cm^3^ in males). This trend highlights the correlation between CAD and EAT volume and its potential role as a predictor of CAD severity. Furthermore, EAT volume was consistently higher in males than females within each CAD-RADS category, and women in the same categories were generally older than men. This observation aligns with the delayed onset of CAD in women, a phenomenon which is often attributed to hormonal and metabolic differences. In summary, while higher EAT volumes are associated with increasing CAC levels in the general population and, particularly, in men, the non-linear pattern observed in women points to more complex interactions that warrant further investigation. These findings emphasize the need for gender-specific considerations when evaluating EAT as a potential biomarker for coronary artery disease.

We also extrapolated a population of subjects who, despite having received clinical indication to perform CCTA, were categorized as CAD-RADS 0, thus being considered healthy for CAD. In this population of patients (n = 120), approximately 24.5% of the patients were free of coronary stenosis and calcifications, and we found the lowest values of EAT volume. The mean EAT volume in the general population was 116 cm^3^, reaching 123 cm^3^ in males and 113 cm^3^ in females; these values align with the proposed normal range for EAT volume in those considered healthy for CAD (<125 cm^3^).

These results are in line with the current literature, which identifies 125 cm^3^ as the threshold to consider the EAT volume high since it is related to adverse cardiovascular events; therefore, the EAT volume values in the population considered healthy for CAD could be considered normal [30,31].

The correlation between EAT volume and the CAD-RADS categories was the real novelty introduced by our study. Since the introduction of CAD-RADS in 2015, several studies have confirmed its role in predicting the onset of MACE and adverse outcomes, but no one has ever correlated EAT volume values with CAD-RADS categories [32].

Kato et al. studied the data obtained from 13 studies concerning approximately 37,595 CCTA exams and demonstrated how all categories except for CAD-RADS 1 could predict all-cause mortality. Moreover, they demonstrated how the combination of CAD-RADS with traditional cardiovascular risk factors and with CAC could increase the predictive capacity of adverse events of CCTA [33].

Precisely for these reasons, we tried to find a link among CAD-RADS 2.0 categories, CAC, and EAT volume, which can also be considered an imaging cardiovascular biomarker.

We also demonstrated a statistically significant positive correlation between the EAT volume and the CAD-RADS categories from 0 to 5 in patients who had already been revascularized (modifier S) and in patients with plaques showing characteristics of vulnerability (modifier HRP). Moreover, in each studied category, we found a clear dichotomy between mean EAT volume values expressed in cm^3^ in female and male patients, with the latter always showing greater amounts of adipose tissue. The volumes in males are always greater than in women and increase with age [34].

We noticed that, within the same categories, women’s age was always greater than that of men. These data are particularly interesting, especially in line with the different age incidence of CAD which affects women later than men [35,36].

The presence of higher EAT values in those with higher CAC further supports its potential role as a cardiovascular imaging marker. Patients with a greater amount of coronary calcification typically face a greater risk of coronary artery disease death or non-fatal myocardial infarction compared to those without calcifications, as demonstrated by several studies [12]. As it is possible to measure CAC and EAT volume through non-contrast CT, it may be possible to combine these two parameters to have a more accurate estimate of cardiovascular risk also in those who cannot receive intravenous iodinated contrast medium [15].

Nowadays, many studies have supported the growing role of CAC as an imaging cardiovascular risk biomarker. CAC is considered the most sensitive coronary heart disease (CHD) screening tool in asymptomatic diabetic patients [37], and, recently, the National Lung Screening Trial has demonstrated the prognostic role of Agatston scoring for cardiovascular death risk also on non-gated non-contrast chest CT scans [38].

For these reasons, the SCCT guidelines recommend (class I) to assess, on a visual scale, CAC as none, mild, moderate, or severe [39].

Interestingly, our study demonstrates a direct correlation between EAT volume and CAC from 0 to 1000. In fact, in our cohort of patients, we found a direct correlation between increasing values of coronary calcium and both higher CAD-RADS categories (CAD-RADS 1-5) and larger EAT volumes. A higher peak in EAT volume and CAD-RADS categories in the P3 category, i.e., in those with a calcium score between 301 and 999, was demonstrated. Furthermore, linearity was interrupted in the category considered to be at greater risk, P4, precisely because of the altered metabolism that affects not only the cardiovascular risk but also all-cause mortality.

These results are in accordance with Peng et al., who evaluated patients with extensive CAC (CAC ≥1000), demonstrating that subjects with CAC > 300 or >400 are usually considered the highest risk group for CVD, while individuals with CAC ≥1000 should be considered a separate risk category that may benefit from the most intensive preventive therapies. They highlighted a continued logarithmic increase in CVD, CHD, cancer, and all-cause mortality without clear evidence of a risk plateau in this group of patients [40].

Several potential limitations of our study should also be addressed. Firstly, the monocentric and retrospective design may limit the generalizability of the results and introduce potential biases related to data collection and interpretation. Moreover, our study was conducted in the absence of healthy individuals to estimate normal EAT volume values. It would be beneficial to measure the volume of EAT in a cohort of truly healthy individuals to establish a more robust baseline for comparison. However, our study cohort included patients classified as CAD-RADS 0 and having a CAC score of 0 who can be considered healthy individuals for CAD. Additionally, we had limited access to detailed data on ongoing pharmacological therapies and major adverse cardiac event (MACE) incidence during the period following CCTA, a fact which may influence the results and reduce the ability to fully assess their impact. In fact, an elevated EAT volume is directly associated with the severity of coronary stenosis and serves as an independent predictor of MACE, including myocardial infarction (MI) and cardiovascular death, independently of other conventional risk factors such as gender and age [7,41]. This can undoubtedly represent an interesting starting point to be explored further with another study, possibly of a prospective nature. Another limit is linked to the definition of EAT: in current literature, pericardial fat and epicardial fat are often used interchangeably and this may influence the comparison of results, as the proximity between visceral and parietal pericardium could lead to inaccurate measurement [30]. Nonetheless, a good part of the works uses the definition proposed by Iacobellis et al. [28] that we also applied in our study. The difference between the two tissues is, therefore, substantial, as there is both a functional connection and an anatomical contiguity between the EAT and the coronary arteries and with the myocardium that does not exist with the pericardial adipose tissue [25]. Moreover, although the CCTA-based EAT measurement is a reproducible method and its feasibility is widely validated in the literature, radiation exposure could be a concern. However, the introduction of new scans such as photon-counting detector CT and the implementation of low-dose protocols could lead to a substantial reduction in the radiation dose. Furthermore, this limitation can be overcome by cardiac magnetic resonance (CMR) imaging, which is considered a feasible radiation-free tool for EAT volume measurement [42,43].

Lastly, the duration of semi-automatic measurements can be time consuming: this limit can be partially overcome by automatic measurements that appear to be as accurate as semi-automatic ones based on data obtained from recent studies [44].

## 5. Conclusions

In conclusion, our study demonstrates a direct correlation between the volume of epicardial adipose tissue (EAT) and factors such as sex, age, CAD-RADS categories, and coronary calcium score. We observed a progressive increase in EAT volume corresponding to greater severity of CAD and stenosis, with notable differences between men and women. A mean EAT volume value of 149 cm^3^ was measured in the CAD-RADS 5 category, with higher values in the male population (151 cm^3^) and lower volume values highlighted in those individuals considered healthy for CAD, i.e., individuals categorized as CAD-RADS 0.

These findings align with the existing literature, confirming the feasibility of cardiac computed tomography angiography (CCTA) for measuring cardiac EAT volume. Furthermore, our results reinforce the observation that higher EAT volumes are associated with more severe coronary artery disease. Notably, this is the first study to establish a correlation between the EAT volume and CAD-RADS categories, confirming their growing importance as imaging cardiovascular biomarkers. However, further research is required to validate these findings and expand upon their clinical implications.

## Figures and Tables

**Table 1:** Clinical and demographic characteristics of the study cohort (n = 489).

Features	Values
No. of patients (n)	489
Men (n)	275 (56.2%)
Female (n)	214 (43.8%)
Age (y), median (IQR)	64 (16)
Age women (y), median (IQR)	67 (15)
Age men (y), median (IQR)	63 (16)
Body mass index, median (IQR)	26 (4)
EAT volume (cm^3^), median (IQR)	133 (73)
- women	124 (68)
- men	142 (83)
Calcium Agatston score (median, IQR)	60 (324)
Smoker, n (%)	179 (36.6%)
Diabetes mellitus, n (%)	144 (29.4%)
High blood pressure, n (%)	257 (52.6%)
Dyslipidemia, n (%)	218 (44.6%)
Family history of heart disease, n (%)	108 (22.1%)

Non-parametric variables are expressed as the median and IQR, categorical variables as absolute values (n) and percentages (%). Abbreviation—y: years; IQR: interquartile range; cm^3^: cubic centimeters.

**Table 2:** Distribution of patients across CAD-RADS categories along with epicardial adipose tissue (EAT) volume statistics. The table presents the number of patients, gender distribution, and median EAT volume (cubic centimeters) with interquartile ranges (IQR) for the general population and stratified by sex (women and men).

CADRADS Categories	No. of Patients (%)	No. of Women	No. of Men	Median EAT Volume (IQR)	Median EAT Volume (IQR) in Women	Median EAT Volume (IQR) in Men
**0**	120 (24.5%)	67 (55.9%)	53 (44.1%)	116 cm^3^ (85 cm^3^)	113 cm^3^ (72 cm^3^)	129 cm^3^ (97 cm^3^)
**1**	93 (19%)	45 (48.4%)	48 (51.6%)	139 cm^3^ (64 cm^3^)	124 cm^3^ (52 cm^3^)	139 cm^3^ (90 cm^3^)
**2**	100 (20.4%)	42 (42%)	58 (58%)	145 cm^3^ (63 cm^3^)	139 cm^3^ (64 cm^3^)	140 cm^3^ (71 cm^3^)
**3**	56 (11.5%)	21 (37.5%)	35 (62.5%)	143 cm^3^ (75 cm^3^)	137 cm^3^ (71 cm^3^)	147 cm^3^ (71 cm^3^)
**4a and 4b**	92 (18.8%)	34 (37%)	58 (63%)	139 cm^3^ (71 cm^3^)	141 cm^3^ (59 cm^3^)	145 cm^3^ (76 cm^3^)
**5**	28 (5.7%)	5 (17.9%)	23 (82.1%)	149 cm^3^ (72 cm^3^)	142 cm^3^ (72 cm^3^)	151 cm^3^ (72 cm^3^)
**Modifier HRP**	42 (8.6%)	12 (28.6%)	30 (71.4%)	142 cm^3^ (71 cm^3^)	134 cm^3^ (72 cm^3^)	146 cm^3^ (72 cm^3^)
**Modifier S**	90 (18.4%)	22 (24.4%)	68 (75.6%)	133 cm^3^ (62 cm^3^)	131 cm^3^ (61 cm^3^)	137 cm^3^ (69 cm^3^)

**Table 3:** Median and interquartile range (IQR) of epicardial adipose tissue (EAT) volume across different coronary artery calcium (CAC) categories in the general population, women, and men. The CAC categories are defined as 0, P1 (1–100), P2 (101–300), P3 (301–999), and P4 (>1000).

CAC (Agatston) (Plaque Burden Category)	Study Cohort (n) Median EAT Volume cm^3^ (IQR cm^3^)	Women Cohort (n %) Median EAT Volume cm^3^ (IQR cm^3^)	Men Cohort (n %) Median EAT Volume cm^3^ (IQR cm^3^)
**0**	126 123 (61)	71(56.34%) 117 (57)	55 (43.65%) 129 (69)
**1–100 (P1)**	100 137 (77)	60 (60%) 131 (75)	40 (40%) 144 (81)
**101–300 (P2)**	70 143 (65)	25 (35.7%) 147 (60)	45 (64.3%) 140 (73)
**301–999 (P3)**	66 143 (69)	29 (44%) 140 (63)	37 (56%) 145 (76)
**>1000 (P4)**	37 145 (80)	16 (43.2%) 115 (76)	21 (56.8%) 154 (75)

**Table 4:** This table presents the combined results of the univariate linear regression analysis evaluating the relationship between log-transformed epicardial adipose tissue (EAT) volume and various clinical variables, grouped by the presence (CAD = 1) or absence (CAD = 0) of coronary artery disease (CAD).

Variable	Patients Positive for CAD (n = 369) Coefficient (*p*-Value)	Patients Negative for CAD (n = 120) Coefficient (*p*-Value)
**Sex**	0.095 (*p* = 0.061)	0.021 (*p* = 0.814)
**Age**	0.003 (*p* = 0.182)	0.011 (*p* = 0.0001)
**BMI**	−0.011 (*p* = 0.263)	0.165 (*p* < 0.0001)
**CAC**	−0.00004 (*p* = 0.334)	0.0014 (*p* = 0.003)
**Smoking**	0.005 (*p* = 0.920)	−0.826 (*p* < 0.0001)

## Data Availability

The data presented in this study are available upon request from the corresponding author.

## Abbreviations

The following abbreviations are used in this manuscript:

EAT	Epicardial adipose tissue
CCTA	Coronary computed tomography angiography
CAC	Coronary artery calcium
CAD	Coronary artery disease
NCCCT	Non-contrast cardiac CT
CAD-RADS	Coronary artery disease reporting and data system
